# The Impact of COVID-19-Related Lockdown on Diet and Serum Markers in Healthy Adults

**DOI:** 10.3390/nu13041082

**Published:** 2021-03-26

**Authors:** Nives Bogataj Jontez, Karin Novak, Saša Kenig, Ana Petelin, Zala Jenko Pražnikar, Nina Mohorko

**Affiliations:** Faculty of Health Sciences, University of Primorska, 6310 Izola, Slovenia; 97200376@student.upr.si (N.B.J.); karin.novak@fvz.upr.si (K.N.); Sasa.Kenig@fvz.upr.si (S.K.); Ana.Petelin@fvz.upr.si (A.P.); Zala.Praznikar@fvz.upr.si (Z.J.P.)

**Keywords:** lockdown, quarantine, nutrition, physical activity, serum biomarkers

## Abstract

Due to limited data about the impact of lockdown on health status, the present study aimed to investigate the impact of COVID-19-related lockdown on changes in dietary habits, physical activity and serum markers in healthy adults. A total of 38 asymptomatic adults aged from 23 to 59 with a normal BMI (22.5 kg/m^2^) participated in baseline and post-lockdown measurements that included dietary and physical activity assessment, anthropometric measurements and blood samples; and the lockdown survey which included dietary assessment and questionnaires about changes in lifestyle and physical activity. A decreased diet quality during lockdown was observed (Healthy Eating Index reduced from 64.59 to 61.08), which returned to near baseline post-lockdown. Energy intake decreased during lockdown (*p* = 0.002) and returned to baseline post-lockdown. Despite lower physical activity levels during lockdown (*p* = 0.035), we observed no significant changes in body composition. However, we observed a significant increase in serum glucose (*p* = 0.005), total cholesterol (*p* = 0.003), and low-density lipoprotein (LDL) (*p* = 0.049) post-lockdown. Increase in serum glucose levels was pronounced in subjects with higher increase in energy intake (*p* = 0.039), increased omega-6 fatty acids intake (*p* = 0.016), those who were exposed to several risky contacts (*p* = 0.018, compared to those with less risky contacts) and those who were not active in nature (*p* = 0.008, compared to those active in nature). Increased serum LDL was correlated to decreased monounsaturated fatty acids intake (*p* = 0.028). Within the limits of this preliminary report, changes in serum markers observed among healthy subjects point to a possible impact of COVID-19-related lockdown on adults’ health to be confirmed in larger groups.

## 1. Introduction

The ongoing coronavirus disease 2019 (COVID-19) outbreak has led to an unprecedented worldwide public health crisis. The COVID-19 outbreak was first reported in late December 2019 in Wuhan, Hubei Province, China. On 11 March 2020, the World Health Organization (WHO) declared the outbreak of COVID-19 to be a pandemic [1]. By the beginning of April 2020, half of the world population was in quarantine or lockdown that lasted until the end of April 2020 [2]. Isolation is different from lockdown as it is used only for those who are infected or sick [3]. The Slovenian government declared an epidemic status on 12 March 2020 [4] and on 16 March 2020 the gathering of people in public places was restricted and all unessential business was closed. The epidemic status ended on 15 May 2020 [5], but many restrictions remained. Lockdown or social distancing is an effective tool to prevent the spread of a new infectious disease [6], but affects lifestyle, including nutrition and physical activity. Evidence from recent studies suggests that pandemic-related coping strategies may have an adverse impact on health [7].

Behavioural changes occurred in a very short time. Restaurants, cafes, schools and kindergartens, gyms and recreational sport facilities were closed, and many people were working from home. Boredom, feelings of uncertainty, loss of freedom and separation from family and friends might have an impact on the psychological status of a person [3]. The increase in unstructured time, stress, and anxiety can further lead to overeating, sedentary behaviour and body mass gain [8]. Humans are sociable beings, so this period of being separated from family and friends caused a lot of people to eat more in quantity or frequency as a mechanism to cope with the growing fear and anxiety [9]. Fear of food shortage can lead to excessive food purchase and consumption [10] and in fact mass purchases of food took place in Slovenia, which was reported by the local media [11]. Mass purchases of food with a long shelf life, instead of fresh food, vegetables and fruit could lead to gaining body fat mass and a reduced intake of antioxidants. Additionally, lockdown restricted activities such as shopping, walking and playing outdoor games; sport facilities were also closed. As a result, many people avoided physical activity and lived a sedentary life. Insufficient physical activity leads to an increased risk of chronic noncommunicable diseases [3].

Consuming a well-balanced diet, focused on fruits and vegetables, wholegrains, plant and animal protein and unsaturated fatty acids leads to good health and normal immune function [9]. Higher fresh fruit and vegetables intake is linked to a higher intake of dietary fibre, vitamins, minerals and antioxidants. Adequate vitamin intake leads to a better immune defence and antioxidant intake improves low-grade inflammation [12]. Diets rich in antioxidants (such as Mediterranean diet) are associated with a reduction in incidence and prevalence of chronic noncommunicable diseases such as cardiovascular disease, type 2 diabetes mellitus and cancer [10] which also seem to be a risk factor for the COVID-19 critical outcome. It is therefore crucial to maintain a good nutritional status and physical activity during periods with high infection risk.

Obesity, hypertension and insufficient physical activity, higher fasting serum glucose and higher total cholesterol levels have been identified by the WHO Global Health Observatory data as the most common and preventable risk factors for chronic noncommunicable diseases [13]. Increased waist circumference, increased serum triglyceride levels, reduced serum HDL levels, increased blood pressure and increased fasting serum glucose levels present the criteria for clinical diagnosis of metabolic syndrome [14] which is related to an increased risk for cardiovascular disease and type 2 diabetes mellitus.

To date, no studies have yet investigated the effects of lockdown on biochemical variables in healthy subjects. The aim of this study was to investigate changes in dietary habits, physical activity, and serum markers due to COVID-19 lockdown in Slovenia in a group of healthy lean subjects with interest in nutrition.

## 2. Materials and Methods

### 2.1. Study Design

The present study is a substudy of a larger study: The Link Between Diets and Health Indicators (DIETE) that started at the Faculty of Health Sciences, University of Primorska in Izola, Slovenia in December 2019 and was interrupted due to the COVID-19 pandemic. The study protocol was approved by the Slovenian National Medical Ethics Committee (No. 0120-557/2017/4) and was registered on ClinicalTrials.gov (Identifier: NCT04347213).

**Figure 1:**
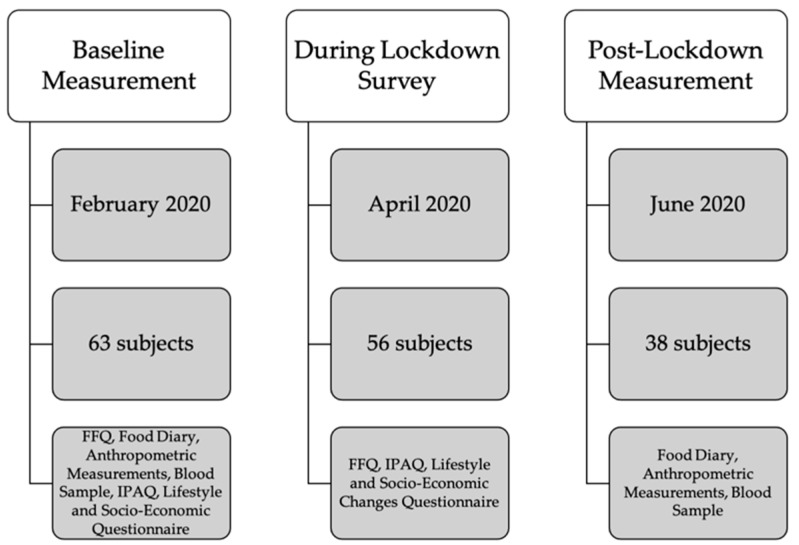
Study Design. FFQ—Food Frequency Questionnaire; IPAQ—International Physical Activity Questionnaire.

Baseline measurement (anthropometric measurements, blood sample, Food frequency questionnaire (FFQ), 3-day Food Diary, Lifestyle Questionnaire, International Physical Activity Questionnaire (IPAQ), Socio-Economic Questionnaire) took place in January, February and the beginning of March 2020 and was then interrupted due to the COVID-19 lockdown. After 4 weeks of lockdown, we invited all subjects with completed baseline measurement to participate in a survey on the effects of lockdown on their lifestyle (FFQ, IPAQ, Lifestyle and Socio-economic Changes Questionnaire). In the beginning of June 2020, they were invited to participate in a post-lockdown measurement (anthropometric measurements, blood sample, 3-day Food diary). All subjects were subjected to the same quarantine rules determined for the whole Slovene population that severely limited individual mobility, obliged everybody to work from home or refrain from work except from certain jobs, listed in the law as exceptions due to their importance for national security. Figure 1 shows the study design.

### 2.2. Study Subjects

Healthy lean volunteers with an interest in nutrition were recruited through a web survey posted on social media in groups dedicated to specific diet or healthy nutrition in December 2019. The inclusion criteria were body mass index (BMI) between 18.5 kg/m^2^ and 30.0 kg/m^2^, and age between 20 and 60 years. The exclusion criteria were any chronic disease, taking medications, being pregnant or lactating, a changed eating pattern 6 months preceding the inclusion in the study and a change in body mass (more than 3 kg) 3 months preceding the inclusion in the study.

A total of 63 adults participated in the baseline measurement before the study was interrupted due to the pandemic. A total of 56 subjects agreed to take part in the lockdown survey. A total of 38 subjects agreed to participate in the post-lockdown measurements, 18 dropped out of the study, mainly for the fear of infection with SARS-CoV-2 while taking part in the measurements at our faculty.

### 2.3. Dietary Assessment

Subjects completed the validated FFQ for the Slovene population [15] and a questionnaire about the food-intake related habits at the baseline and four weeks into lockdown. The FFQ is a retrospective method for evaluating dietary intake in the last four weeks [15] and includes nine food groups: milk and dairy products, vegetables, fruit, starchy foods, legumes, meat and meat products, fat and fatty foods, sugar and beverages. It consists of eight frequency measures (never, once per month, 2 to 3 times per month, 1 to 2 times per week, 3 to 4 times per week, 5 to 6 times per week, 1 to 2 times per day, 3 or more times per day) and 3 portion sizes (small, medium, large). The subjects were provided with visualisation tools for more accurate portion assessment.

Subjects also completed a 3-day food diary at the baseline and post-lockdown. The subjects were instructed to record their food intake for three days in the same week (two weekdays and one weekend day). Where possible, subjects were asked to include food labels and recipes for mixed dishes. They were taught to weigh and record all food and beverages immediately before eating, and to weigh and describe any leftovers. They were also asked to report all food supplements taken that day.

Dietary data from the FFQ and food diary (energy, macronutrient, micronutrient intake and total- oxidative radical absorbance capacity (ORAC)) were analysed with the Open Platform for Clinical Nutrition (OPEN, http://opkp.si/, accessed on 15 May 2020), a freely accessible online dietary assessment and planning tool that includes data from Slovenian nutrition tables [16], Souci Fachmann Kraut database [17] and United States National Nutrient Database for Standard Reference, available through the website http://www.ars.usda.gov/Services/docs.htm?docid=8964, accessed on 15 May 2020.

Diet quality was evaluated by calculating the Healthy Eating Index 2015 (HEI) [18] according to the developer’s protocol [19]. The HEI includes 13 categories: total fruits, whole fruits, total vegetables, greens and beans, whole grains, dairy, total protein foods, seafood and plant protein foods, fatty acids ratio (polyunsaturated and monounsaturated fatty acids/saturated fatty acids), refined grains, sodium, added sugars and saturated fats, all estimated per energy intake unit (1000 kcal). The first nine categories are scored positively and the last four categories are scored negatively from zero to five or to ten points proportionally to the interval between standards, as specified by Center for Nutrition Policy and Promotion [19]. The HEI (0–100) is the sum of points from all categories with higher score representing a higher diet quality.

The HEI was calculated from food diary data on baseline and post-lockdown and from FFQ during lockdown. To evaluate whether the HEI calculated from food diary and FFQ could be compared, HEI validation was performed using FFQ and food diary data at baseline from 17 subjects. Normal data distribution was determined with the Shapiro–Wilk test. Pearson correlation was used to establish the correlation between both methods (r = 0.674, *p* = 0.003). Student’s T-test did not show any statistically significant differences between the two methods (*p* = 0.914), which implies both methods are equally reliable for HEI evaluation.

### 2.4. Lifestyle and Socio-Economic Questionnaire

At baseline, subjects were asked to report their education, marital status, work, socioeconomic status, living arrangement, and smoking and alcohol consumption habits. As part of the lockdown survey, they were inquired about the changes in previously reported data during lockdown, and about the changes in working conditions and workload. To assess the impact of the pandemic on their quality of life, they were asked about the frequency of following the news, whether they perceived changes in lifestyle and quality of life during lockdown, how frequently they visited the grocery store and about the changes of outdoor activities. We also inquired whether they respected social distancing and hygiene measures adopted by the government and whether they contracted COVID-19. 

### 2.5. Physical Activity Questionnaire

The IPAQ [20] was used to calculate the physical activity-induced energy expenditure. The IPAQ includes questions about work-related physical activity, activity due to transport and physical activity during free time. Each question consists of the duration and intensity of the activity. Data from the duration and intensity were used to calculate daily physical induced energy expenditure in the metabolic equivalent of the task (MET).

### 2.6. Anthropometric Measurements

Anthropometric measurements were performed at baseline and post-lockdown following an overnight fast (at least 12 h) in standardized conditions with light clothing and without shoes by the same examiner. Body mass, body fat percentage, fat mass, lean mass, muscle mass, visceral index, total body water, extracellular water, intracellular water and phase angle were measured using a bioelectric impedance analyser Tanita BC 418MA (Tanita Corporation, Arlington Heights, IL, USA).

### 2.7. Serum Markers

Venous blood samples were collected in 5 mL serum vacuum blood collection tubes at baseline and post-lockdown following an overnight fast. Samples were set to clot at room temperature for 30–60 min in line with Standard operating procedures for serum and plasma collection [21] and then centrifuged at 2000 rpm for 10 min on room temperature. Serum was immediately separated, frozen and stored at −80 °C until subsequent analysis. Serum glucose, triglycerides, total cholesterol, low-density lipoprotein (LDL), high-density lipoprotein (HDL), iron, aspartate transaminase (AST), bilirubin and C-reactive protein (CRP) levels were determined with a Cobas c111 analyser (Roche, Basel, Switzerland) using the specific Cobas c111 reagent for each parameter (Roche). Total cholesterol/HDL ratio was calculated for each individual.

### 2.8. Statistical Analysis

Statistical analysis was performed using IBM SPSS Statistics 26.0 (IBM, Armonk, NY, USA). Means and standard deviations were calculated. The normality of data distribution was evaluated using the Shapiro–Wilk test. Student’s paired samples t-test or one-way ANOVA were used to compare the effect of lockdown between two or more normally distributed variables, respectively, while two or more non-normally distributed variables were compared with Wilcoxon’s signed-rank test or Kruskall–Wallis’ test, respectively. Repeated measures ANOVA was used to investigate changes in diet quality from baseline through lockdown to post-lockdown for normally distributed data, for non-normally distributed data Friedman’s test was used. Changes of post-lockdown data from baseline (∆) for nutritional intake and serum markers were calculated by subtracting baseline values from post-lockdown values. Pearson’s Correlation was used to investigate associations between ∆ nutritional intake and ∆ serum markers for normally distributed data, for non-normally distributed data Spearman’s Correlation was used. *p*-values < 0.05 were considered statistically significant.

Post hoc, statistical power of the study was calculated based on primary outcome, ∆ serum glucose, using G*Power 3.1.9.7 (Heinrich-Heine-Universität Düsseldorf, Germany), assuming an α level of 5% and β level of 20%. Effect size for N = 38 was calculated.

## 3. Results

### 3.1. Subjects’ Baseline Characteristics

Subjects’ baseline characteristics are reported in Table 1. The final sample included 14 male and 24 female Caucasian subjects, aged from 23 to 59 years with a normal BMI. The subjects were healthy, asymptomatic adults with stable body mass and stable eating pattern in the last 6 months. A total of 83.8% of the subjects had at least a bachelor’s degree. The majority (81.1%) of the subjects were married or in a non-marital partnership and only 10.8% of the subjects were living alone. A total of 67.6% of the subjects never smoked, while only 16.2% of them never drank alcohol. A total of 55.3% of the subjects consumed meat, while 44.7% of them consumed a plant-based diet. The analysis did not show any statistically significant differences in diet quality between meat-eaters and nonmeat eaters.

### 3.2. Lifestyle and Socio-Economic Changes during Lockdown

Table 2 reports the lifestyle changes during lockdown. Almost all subjects (94.6%) stated that their lifestyle changed during lockdown and more than half (59.5%) of the subjects stated they were following more news than usual. None of the subjects had contracted COVID-19. Only a third of the subjects (33.3%) experienced no changes in working conditions during lockdown, while others were not working or working (at least partially) from home. Almost three quarters (73.0%) of the subjects were active in nature and woods, while others were active only in close proximity to home during lockdown. Of the subjects who were working during lockdown, around half (48.1%) were physically active the same as before during their work time, while others were less active or could not specify an answer.

### 3.3. Changes in Nutrition and Physical Activity during and Post-Lockdown

Subjects consumed on average 3.53 ± 1.03 meals per day at baseline and 3.33 ± 0.96 meals per day during lockdown. The change was not significant (*p* = 0.127).

The HEI dropped significantly during lockdown and improved post-lockdown but did not reach the baseline value (Table 3). Vegetable, greens and beans, protein and seafood and plant protein intake per energy unit and fatty acids intake ratio (polyunsaturated fatty acids [PUFA] + monounsaturated fatty acids [MUFA])/saturated fatty acids [SFA]) significantly decreased throughout the study. In contrast, dairy and refined grains intake per energy unit significantly increased throughout the study. Added sugar intake per energy unit increased during lockdown but decreased post-lockdown. Sodium intake per energy unit was significantly higher post-lockdown. Physical activity induced energy expenditure significantly dropped during lockdown from 13.86 ± 22.48 MET at baseline to 9.89 ± 13.62 MET during lockdown (*p* = 0.035).

Although there were no statistically significant differences between baseline and post-lockdown HEI and energy intake, some significant differences in dietary intake were observed (Table 4). Energy density and omega-6 fatty acids intake significantly decreased, whereas the intake of fibre, saturated fatty acids and starch significantly increased. The intake of some micronutrients, such as riboflavin, niacin, folate, magnesium and copper also increased and total-ORAC also increased post-lockdown.

### 3.4. Anthropometric Characteristics

Subjects reported no changes in body mass during lockdown, which was confirmed in the post-lockdown measurement. There were no statistically significant changes in anthropometric variables post-lockdown (Table 5).

### 3.5. Significant Changes in Serum Markers Post-Lockdown

We observed a statistically significant increase in serum glucose (*p* = 0.005), total cholesterol (*p* = 0.003) and LDL (*p* = 0.049) levels, and a statistically significant decrease in serum CRP levels (*p* = 0.008) post-lockdown (Table 6). Total cholesterol/HDL ratio significantly increased from 2.760 ± 1.171 at baseline to 3.871 ± 3.427 post lockdown (Z = 4.358, *p* < 0.001). Post hoc calculated effect size for N = 38 for ∆ serum glucose (0.361 ± 0.746) was 0.483 and the power of two tailed Student’s test was 0.826.

**Figure 2:**
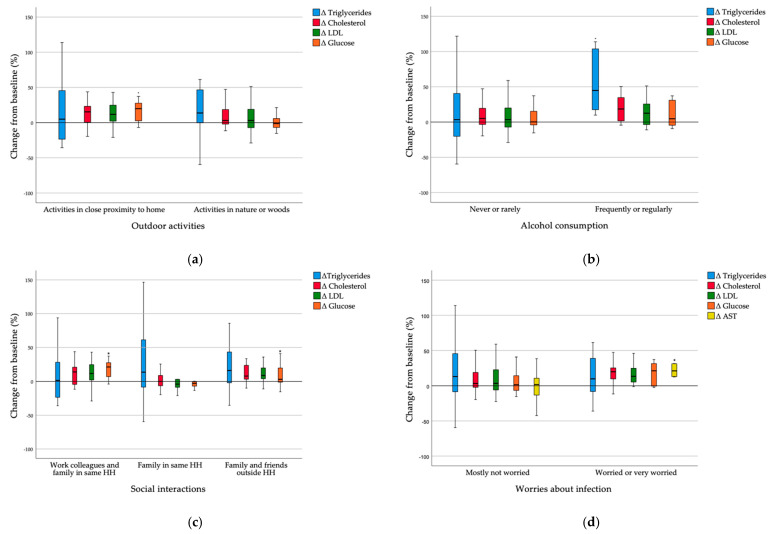
(**a**) Δ serum markers in groups with different outdoor activities during lockdown; (**b**) Δ serum markers in groups with different alcohol consumption during lockdown; (**c**) Δ serum markers in groups with different social interactions during lockdown; (**d**) Δ serum markers in groups with different levels of worries about infection during lockdown. Δ—change of post-lockdown value from baseline; LDL—low-density lipoprotein; AST—aspartate transaminase; HH—household. * *p* < 0.05 is considered statistically significant.

To assess whether the changes in serum markers and dietary intake per energy unit at post-lockdown from baseline differed among different lifestyle circumstances during lockdown, we divided the participants into groups based on reported lifestyle factors during lockdown (Figure 2). Subjects that were active only in close proximity to home had a significantly higher increase in serum glucose levels than subjects that were active in nature or woods (Z = 2.646, *p* = 0.008). A trend of increased serum total cholesterol and LDL levels is also visible, but the changes were not significant (Figure 2a). Subjects that frequently or regularly consumed alcohol during lockdown had a significantly higher increase in serum triglyceride levels than subjects that never or rarely consumed alcohol (Z = −2.417, *p* = 0.016). A trend of increased serum total cholesterol and LDL levels is also visible, but the changes were not significant (Figure 2b). Subjects that were interacting socially with work colleagues or family and friends outside their household had a significantly higher increase in serum glucose levels than subjects who were interacting socially only with family in the same household (χ^2^ = 8.008, *p* = 0.018). A trend of increased serum total cholesterol levels is also visible, but the changes were not significant (Figure 2c). Subjects who were worried or very worried about infection with SARS-CoV-2 had a significantly higher increase in serum AST levels (Z = −2.501, *p* = 0.012) than subjects who were mostly not worried about infection. A trend of increased serum glucose, total cholesterol and LDL levels is also visible, but the changes were not significant (Figure 2d).

Higher vegetable per energy unit intake (*p* = 0.028) and lower dairy (*p* = 0.042) and total protein intake per energy unit (*p* = 0.023) were detected in those who had social interaction only with family in the same household compared to those who had social interactions also outside their household. Furthermore, higher greens and beans per energy unit intake (*p* = 0.037) and lower refined grains intake were found in those who were very worried about infection than in those who were not.

### 3.6. Correlations between Food Quality and Serum Marker Changes Due to Lockdown

**Figure 3:**
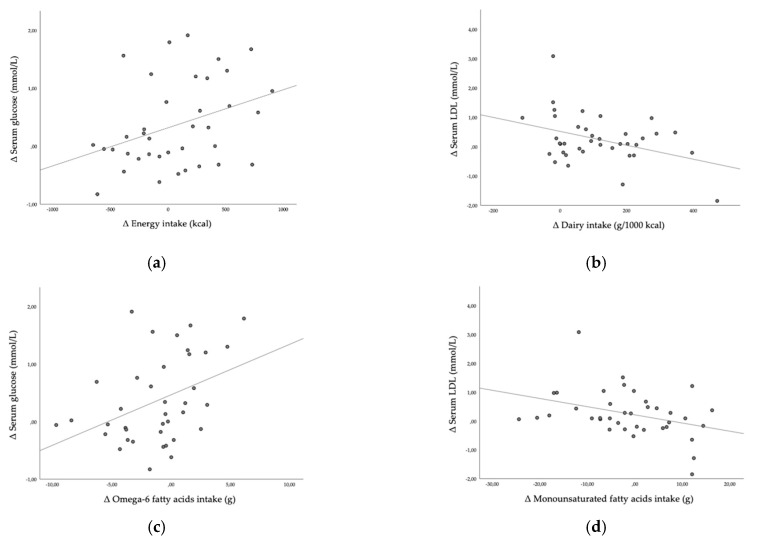
(**a**) Correlation between Δ energy intake and Δ serum glucose; (**b**) correlation between Δ dairy intake and Δ serum LDL; (**c**) correlation between Δ omega-6 fatty acids intake and Δ serum glucose; (**d**) correlation between Δ monounsaturated fatty acids intake and Δ serum LDL; Δ—change of post-lockdown value from baseline.

Furthermore, we investigated how the observed changes in serum parameters were associated to the changes in dietary habits. We observed a statistically significant negative association between ∆ HEI and ∆ serum glucose (r = −0.328, *p* = 0.044). Higher increase in energy intake was positively correlated with higher increase in serum glucose (r = 0.336, *p* = 0.039, Figure 3a). Higher increase in part of energy intake in form of saturated fatty acids was negatively correlated with ∆ serum HDL (r = −0.322, *p* = 0.049), higher increase in dairy intake per 1000 kcal was weakly positively correlated with ∆ serum HDL (r = 0.0372, *p* = 0.022) and negatively correlated with ∆ serum LDL (r = −0.385, *p* = 0.017, Figure 3b) and higher increase in total protein food intake per 1000 kcal was positively correlated with ∆ serum HDL (r = 0.373, *p* = 0.021). Higher increase in omega-6 fatty acids intake was positively correlated with higher increase in serum glucose (r = 0.390, *p* = 0.016, Figure 3c). Higher increase in monounsaturated fatty acids intake was inversely correlated with ∆ serum LDL (r = −0.356, *p* = 0.028, Figure 3d).

## 4. Discussion

This is the first study investigating the impact of COVID-19 lockdown on changes in lifestyle, nutrition and serum markers in healthy adults. Despite a small number of participants, we observed some interesting and potentially important results. There was a transient decrease in diet quality during lockdown, whereas post-lockdown general diet quality returned to near baseline. The subjects reported significantly decreased physical activity during lockdown and a significantly decreased energy intake that returned to baseline post-lockdown. Subjects did not report changes in body mass during lockdown, nor did we observe any change in body composition from baseline in the post-lockdown measurement. Serum markers, however, changed significantly post-lockdown, which may point to a possible impact of lockdown on healthy adults’ health markers.

Blood sample analysis showed a significant increase in glucose, total cholesterol and LDL levels and a decrease in CRP levels post-lockdown in our participants. The mean value of serum CRP levels was below 1 mg/L (the Slovenian reference values are 0–8 mg/L), which is extremely low and the change from baseline to post-lockdown was −0.306 mg/L, which was a minor change, even though it was statistically significant. Total cholesterol and LDL mean values post-lockdown exceeded Slovenian reference values. Higher serum glucose, total cholesterol and LDL levels are risk factors for developing metabolic syndrome [22] and each 1 mmol/L increase in serum cholesterol significantly increases the risk for all-cause mortality in all age groups [23]. A meta-analysis showed the total cholesterol/HDL ratio is the strongest predictor of mortality from ischemic heart disease [24]; the ratio in our study significantly increased from 2.8 to 3.9 (*p* < 0.001), which could implicate a higher risk for heart disease among subjects in the long term. An increase in glucose of 0.361 mmol/L was observed. Increase in serum glucose of 1 mmol/L significantly increased the risk for all-cause mortality in a prospective study and this effect was stronger in younger adults [25].

Higher increases in serum glucose levels were observed in healthy subjects who were worried or very worried about infection with SARS-CoV-2 compared to subjects who were mostly not worried about infection, even though the changes were not statistically significant. Increases in serum glucose levels post-lockdown among subjects that were socially interacting with work colleagues and friends, however, were significantly higher than increases in serum glucose levels in those who were socially interacting only with family in the same household. The subjects who had more social interactions due to work were potentially exposed to a number of risky contacts and therefore were possibly more concerned, experienced more stress and had higher glucose levels. Being worried or experiencing more stress leads to the release of stress hormones such as cortisol, which increases serum glucose levels and influences serum cholesterol levels [26]. We also observed higher increase in serum AST levels among subjects that were worried or very worried about infection with SARS-CoV-2 and some trends for higher increase in serum cholesterol and LDL, but the changes were not statistically significant. Significantly higher increases in serum glucose levels post-lockdown were observed among subjects that were active only in the close proximity to home than among subjects that were active in nature or woods during lockdown. A previous study found that lockdown significantly affected mental health, while contact with nature (blue–green spaces) helped people to cope with these impacts [27], which may explain lower increase in serum glucose levels among subjects that were spending more time in nature.

The literature reports the impact of lockdown on glycaemic control only among patients with diabetes mellitus type 1 and 2. A retrospective study compared glycaemic control of patients with type 1 diabetes mellitus during lockdown to the same time period in the previous year. They found it was significantly worse during lockdown and the use of insulin therapy was significantly higher, which the authors ascribe to the reduced physical activity, changed dietary habits and higher levels of anxiety during lockdown [28]. Similar was found in a study by Barchetta et al. [29]. A study among patients with well-controlled type 2 diabetes mellitus showed worse glucose control during lockdown, which was worse among patients with higher fasting triglyceride levels pre-lockdown [30]. On the contrary, some studies found better glycaemic control in patients with type 1 diabetes mellitus during lockdown, which could be due to having more time for self-management during lockdown, at least in the short term [29,31,32,33] or due to reduced work-related stress [34].

In the present study, we found a decrease in HEI during lockdown. Decreased intake of total protein food, seafood and plant proteins per energy unit, increased intake of refined grains and worse composition of fatty acids in the diet (decreased PUFA+MUFA)/SFA) contributed to this drop. HEI has been associated with lower risk factors for development of chronic noncommunicable diseases, such as cardiovascular diseases, cancer and all-cause mortality [35]. It has also been associated with improved low-grade inflammation [36]. The literature reports different changes in diet quality during lockdown. A small increase in HEI during early lockdown compared with baseline was found in a Canadian study in which the authors hypothesized that eating out less often may explain such an observation [37]. A Spanish survey also observed increased adherence to the Mediterranean diet, which points to a better diet quality during lockdown [38]. On the other hand, one of the Italian surveys did not detect any changes in dietary habits and more than a third of the subjects stated their diet worsened during lockdown [39], while another found a decrease in diet quality [40].

Post-lockdown HEI of our participants returned to nearly baseline levels, but vegetable, greens and beans, total protein and seafood and plant protein intake per energy unit were still significantly lower post-lockdown, while refined grains intake per energy unit and % energy intake in form of saturated fats intake were still significantly higher. Additionally, sodium intake per energy unit increased post-lockdown. Interestingly, the participants who were worried about infection increased vegetable intake per energy unit post lockdown and lowered refined grains per energy unit intake more than those who were not worried about infection. Higher ∆ vegetable intake was observed also in those who had social interaction only with family in the same household compared to those who had social interactions also outside their household. An increase in starch and dietary fibre intake was observed in all participants. Post-lockdown dairy intake per energy unit increased and added sugars intake per energy unit decreased, which contributed to a higher HEI at post-lockdown than during lockdown. We observed a statistically significant negative association between ∆ HEI and ∆ serum glucose. Increase in dairy intake per energy unit positively correlated to ∆ serum HDL and negatively correlated to ∆ serum LDL, which is in line with reported positive association between dairy intake and lipid profile [41]. We also observed a correlation between increase in total protein food per energy unit intake and ∆ serum HDL. The ratio of fatty acids intake worsened and saturated fatty acids intake and saturated fatty acids per energy unit were significantly higher post-lockdown. It is known that an increase in saturated fatty acids intake is associated with increased serum total cholesterol and LDL [42]. In our study, we observed an increase in serum total cholesterol and LDL, but the associations between ∆ serum total cholesterol and ∆ serum LDL and ∆ saturated fatty acid intake were not statistically significant; however, higher increase in saturated fatty acids per energy unit was significantly negatively correlated with ∆ serum HDL. It has been shown that an increase in saturated fatty acids intake could lead to a temporary insulin resistance and consequently higher serum glucose levels [43]. An increase in serum glucose was indeed observed, but there were no associations between ∆ serum glucose and ∆ saturated fatty acid intake. We observed a negative association between ∆ monounsaturated fatty acids intake and ∆ LDL. The intake of omega-6 fatty acids decreased and consequently the omega-3/omega-6 fatty acids ratio increased. Despite this, we observed a statistically significant association between ∆ omega-6 fatty acids intake and ∆ serum glucose. It should be noted, however, that the aforementioned correlations were weak to moderate.

Energy intake decreased during lockdown, but no changes in energy intake between baseline and post-lockdown were observed, even though there was a decrease in energy density. Our participants also maintained a constant number of meals during lockdown. Nevertheless, there was a statistically significant association between increased energy intake and increased serum glucose. Contrary to our study, other studies reported an increased energy intake during lockdown [44], an increased number of meals per day [45] and increased consumption of homemade pastries and fried food [46], which may lead to increased energy intake [47]. An increased snacking [46], especially in sweet snacks intake was also observed [44,45,48]. Moreover, studies also reported an increase in snacking between meals and late-night snacking, especially an increase in the intake of salty snacks [45,49]. In one study, almost half of the subjects felt anxious about their eating habits and stated they consumed more food, particularly comfort foods with the aim to feel better [50]. We observed a trend in increased alcohol intake during lockdown that was not statistically significant (data not shown) but did not observe any changes in alcohol intake between post-lockdown and baseline. This is in line with a previous report, where 70% of the subjects reported no changes in their alcohol intake during lockdown [46]. However, significantly higher increase in serum triglyceride levels was observed among subjects that frequently or regularly consumed alcohol during lockdown than those that never or rarely consumed alcohol, pointing to a known fact that excessive alcohol intake may cause hypertriglyceridemia postprandially and in the fasting state [51]. An increase in alcohol intake was observed in some other studies [44,48].

The subjects’ physical activity levels were lower during lockdown, which was reported also in other studies [39,40,41,52]. One study [44] reported increased screen time in half of the subjects, which could be one reason for lower physical activity levels; other reasons include closed sport facilities, restricted outdoor activities and less physical activity due to work and transport. An online survey also found more than a third of the subjects did not engage in any form of physical activity and they also reported five or more hours of screen time per day [53]. On the contrary, an Italian survey reported no changes in physical activity during lockdown [40]. Decreased physical activity itself is a risk factor for low-grade inflammation and development of metabolic disorders [54]; however, despite lower physical activity levels, we did not observe any increase in inflammatory marker CRP. The observed increase in fasting serum glucose could be partially due to decreased physical activity, as the association was shown before [55].

Despite lower physical activity levels during lockdown, we did not observe any significant changes in body mass or body composition, probably due to a lower energy intake during lockdown. Maintenance in body mass in our study is contrary to most other studies, which reported an increase in body mass during lockdown [37,46,53,56,57], although there are also studies with observations similar to ours in patients with type 2 diabetes mellitus [58], and in healthy subjects [40]. Interestingly, it was observed that overweight and obese subjects gained body mass, while underweight subjects lost it [48]. Our subjects all had normal body mass at baseline.

There are limitations to our study. The sample size (38 subjects) is small. The subjects dropped-out because of their fear of infection with SARS-CoV-2 while taking part in the measurements at our faculty. While the sample was big enough for evaluating changes in serum parameters (statistical power of primary outcome, ∆ serum glucose was 0.826), a bigger sample would be needed to thoroughly evaluate changes in lifestyle. Due to lockdown restrictions, we could not perform lockdown measurement in our facility, therefore for that timepoint we only have data that could be collected online with questionnaires. The subjects were healthy volunteers who had an interest in nutrition and were already engaging in healthy behaviours, so the sample may not be representative for the general population. Nevertheless, our results provide some insight into the impact of lockdown on health markers in asymptomatic subjects with normal body mass, which was very scarce before.

Social distancing and psychological stress due to the COVID-19 pandemic have been reported to have led to harmful health behaviours that are associated with noncommunicable diseases and can interfere with immunity [59]. Moreover, managing lifestyle risk factors, such as diet, physical activity, smoking and alcohol consumption can prevent severe COVID-19 outcomes [60]. Impaired glucose control and dyslipidaemia have also been demonstrated to be risk factors for worse COVID-19 outcomes [61,62,63]. We observed a worsened serum biomarkers profile after lockdown among healthy subjects with a normal body mass, healthy lifestyle and eating patterns. Despite observed changes in diet quality, changes in serum biomarkers could not be explained by them. Our participants maintained similar energy intake post-lockdown to background, their omega-6 fatty acids intake decreased, while total protein food intake per energy unit and dairy intake increased. Observed moderate correlations between aforementioned changes in nutrition and changes in serum markers point to a possible small positive effect of nutritional changes on serum markers. There was, however, an increase in serum glucose, total cholesterol and LDL post-lockdown. Furthermore, we observed increased dietary fibre and total ORAC intake and decreased added sugar intake, which have all been reported to have favourable effects on serum glucose and cholesterol [64]. Increase in serum glucose with the duration of confinement was reported in a study of 520-day mission simulation to Mars [65]. Effects of factors such as worry, stress and confinement on serum markers could be taken into account for the observed changes in serum markers. The fact that the improvement of certain healthy behaviours observed in our small study did not counterbalance the effect of stress is an important public health aspect that should be further explored.

## 5. Conclusions

This is the first study investigating the changes in diet and physical activity during lockdown and their effect on serum markers in healthy adults. Significant changes in serum markers were observed after only 3 months of lockdown, such as an increase in serum glucose, total cholesterol, and LDL levels. Such changes increase the risk for developing metabolic syndrome, heart disease and all-cause mortality. The changes of serum markers therefore point to a possible impact of lockdown on health markers.

## Figures and Tables

**Table 1:** Study Subjects’ Baseline Characteristics.

Characteristics	Mean ± SD
n	38
Age (years)	36.3 ± 10.1
Height (cm)	170.8 ± 8.3
Body mass (kg)	65.8 ± 10.2
BMI (kg/m^2^)	22.5 ± 2.7
**Gender**	**%**
Female	63.2
Male	36.8
**Education**	**%**
High school diploma	16.2
Bachelor’s degree	70.3
Master’s degree or PhD	13.5
**Marital Status**	**%**
Single	18.9
In a relationship or married	81.1
**Living Arrangement**	**%**
Alone	10.8
With partner	35.1
With partner and children	40.5
With parents	13.5
**Smoking**	**%**
Never	67.6
Occasionally	24.3
Regularly	8.1
**Alcohol Consumption**	**%**
Never	16.2
Occasionally	75.7
Regularly	5.4
**Meat Consumption**	**%**
Regular meat consumption	55.3
No meat consumption	44.7

**Table 2:** Lifestyle Changes During Lockdown.

**General Lifestyle Changes during Lockdown**	**%**
Lifestyle did not change at all	5.4
Lifestyle changed a little	56.8
Lifestyle changed a lot	32.4
Lifestyle changed completely	5.4
**Changes in working conditions during lockdown**	**%**
No changes	33.3
Work from home, previously partially worked from home	11.1
Work from home, previously never worked from home	13.9
Partially work from home and partially at the workplace	11.1
Not working, receiving compensation	22.2
Not working, not receiving compensation	8.3
**Changes in socio-economic status during lockdown**	**%**
Received 0–50% of baseline monthly income	16.2
Received 80% of baseline monthly income	16.2
Received 90–100% of baseline monthly income	67.6
**Visiting the grocery store during lockdown**	**%**
Less than once per week	35.1
Once per week	48.6
Twice per week	13.5
More than twice per week	2.7
**Activities outdoors during lockdown**	**%**
Activities in close proximity to home	27.0 %
Activities in the nature and woods	73.0 %

**Table 3:** Energy Intake and Healthy Eating Index and its Categories at Baseline, During Lockdown and Post-Lockdown.

	Baseline	Lockdown	Post-Lockdown	*p* Value *
Energy intake (kcal)	2296.78 ± 873.26	1891.03 ± 678.13 ^a^	2248.17 ± 576.76 ^c^	0.002
Total fruits (g/1000 kcal)	109.79 ± 102.00	113.57 ± 101.45	108.75 ± 67.38	0.916
Whole fruits (g/1000 kcal)	85.77 ± 78.25	90.51 ± 78.49	83.13 ± 62.66	0.829
Total vegetables (g/1000 kcal)	507.16 ± 377.07	581.21 ± 411.26	134.05 ± 92.13 ^b,c^	<0.001
Greens and beans (g/1000 kcal)	76.57 ± 113.65	54.04 ± 57.01	22.39 ± 23.31 ^b,c^	0.002
Whole grains (g/1000 kcal)	23.14 ± 24.75	21.11 ± 24.41	28.85 ± 29.30	0.319
Dairy (g/1000 kcal)	60.70 ± 69.39	64.65 ± 67.59	172.98 ± 143.27 ^b,c^	<0.001
Total protein food (g/1000 kcal)	88.95 ± 66.92	69.31 ± 46.38 ^a^	54.55 ± 33.47 ^b^	0.002
Seafood and plant proteins (g/1000 kcal)	49.27 ± 39.97	35.37 ± 28.92 ^a^	29.91 ± 22.52 ^b^	0.002
Fatty acids (PUFA+MUFA)/SFA)	1.98 ± 1.34	1.77 ± 1.20 ^a^	1.54 ± 0.78 ^b^	0.026
Refined grains (g/1000 kcal)	29.67 ± 28.17	39.81 ± 37.58 ^a^	52.72 ± 48.19 ^b^	0.006
Sodium (g/1000 kcal)	0.95 ± 0.44	1.12 ± 0.73	1.19 ± 0.54 ^b^	0.071
Added sugar (%EI)	10.93 ± 6.08	12.39 ± 6.67	4.84 ± 3.93 ^b,c^	<0.001
Saturated fats (%EI)	10.98 ± 7.46	11.82 ± 8.47	13.23 ± 8.79 ^b,c^	0.001
HEI	64.59 ± 15.76	61.08 ± 13.42 ^a^	63.26 ± 15.22	0.203

HEI—Healthy Eating Index. *—repeated measures ANOVA or Friedman’s test. ^a^—*p* < 0.05, Student’s or Wilcoxon’s signed-rank test between baseline and lockdown; ^b^—*p* < 0.05, Student’s or Wilcoxon’s signed-rank test between baseline and post-lockdown; ^c^—*p* < 0.05, Student’s or Wilcoxon’s signed-rank test between lockdown and post-lockdown measurement.

**Table 4:** Energy, Macronutrient and Micronutrient Intake from Diet at Baseline and Post-Lockdown.

Variable (Unit)	Baseline	Post-Lockdown	*p* Value *
Energy density (kcal/g) *	1.15 ± 0.32	0.81 ± 0.27	0.000
Protein (g)	86.47 ± 36.45	87.25 ± 36.45	0.567
Carbohydrate (g)	241.18 ± 151.18	249.45 ± 131.06	0.456
Fat (g)	89.29 ± 35.63	93.01 ± 41.82	0.345
Dietary fibre (g) *	28.35 ± 17.30	31.49 ± 17.95	0.046
Dietary fibre (g/1000 kcal) *	12.81 ± 6.45	14.15 ± 7.82	0.031
Soluble fibre (g)	5.54 ± 4.49	6.02 ± 3.84	0.272
Unsoluble fibre (g)	10.93 ± 8.70	12.23 ± 8.18	0.197
Sugar (g)	80.62 ± 55.72	76.70 ± 48.44	0.690
Free sugar (g)	33.53 ± 29.16	29.70 ± 27.88	0.464
Starch (g) *	68.13 ± 62.95	84.17 ± 58.09	0.048
Plant protein (g)	37.26 ± 24.89	40.82 ± 24.46	0.065
Saturated fatty acids (g) *	26.54 ± 18.82	31.76 ± 20.68	0.003
Monounsaturated fatty acids (g)	28.55 ± 14.23	27.07 ± 12.73	0.378
Polyunsaturated fatty acids (g)	13.25 ± 7.84	12.43 ± 6.10	0.421
Omega-6 fatty acids (g) *	7.29 ± 5.95	6.05 ± 4.26	0.042
Omega-3 fatty acids (g)	1.45 ± 1.59	1.50 ± 1.26	0.357
Omega-3/omega-6 ratio *	0.27 ± 0.39	0.29 ± 0.18	0.023
Cholesterol (mg)	323.35 ± 376.50	316.96 ± 356.47	0.328
Vitamin C (mg)	135.40 ± 99.23	110.94 ± 70.30	0.232
Vitamin D (μg)	4.08 ± 3.45	4.61 ± 5.08	0.637
Vitamin E (mg)	12.51 ± 7.72	10.83 ± 4.78	0.255
Riboflavin—vitamin B_2_ (mg) *	1.65 ± 0.62	2.00 ± 0.76	0.003
Niacin—vitamin B_3_ (mg) *	20.10 ± 10.99	29.56 ± 12.26	0.000
Biotin—vitamin B_7_ (μg)	35.78 ± 20.41	38.97 ± 19.10	0.076
Folate (μg) *	360.91 ± 175.88	442.64 ± 225.83	0.001
Vitamin B_12_ (μg)	5.02 ± 6.62	5.70 ± 13.54	0.983
Calcium (mg)	855.04 ± 373.67	938.57 ± 468.35	0.421
Iron (mg)	17.23 ± 7.38	18.24 ± 6.55	0.210
Magnesium (mg) *	407.79 ± 224.65	560.13 ± 224.44	0.000
Zinc (mg)	10.56 ± 5.35	11.54 ± 4.46	0.062
Copper (μg) *	1865.12 ± 923.61	2429.81 ± 1144.20	0.001
Alcohol (g)	6.41 ± 11.96	5.88 ± 10.02	0.387
Total-ORAC (μmol/TE) *	8346.48 ± 6271.20	10874.57 ± 6949.49	0.005

*—*p* < 0.05; Total-ORAC—total oxidative radical absorbance capacity of daily food intake.

**Table 5:** Anthropometric Variables of Asymptomatic Subjects at the Baseline and Post-Lockdown.

	Baseline	Post-Lockdown	*p* Value
Body mass (kg)	65.8 ± 10.2	66.3 ± 10.5	0.342
Body fat percentage (%)	22.2 ± 7.6	22.4 ± 7.2	0.639
Fat mass (kg)	14.7 ± 5.5	14.8 ± 5.2	0.711
Fat free mass (kg)	51.1 ± 8.9	51.4 ± 9.6	0.400
Muscle mass (kg)	48.5 ± 8.5	48.8 ± 9.1	0.442
BMI (kg/m^2^)	22.5 ± 2.7	22.7 ± 2.6	0.413
Visceral index	3.8 ± 2.3	3.7 ± 2.3	0.059
Total body water (kg)	36.6 ± 6.3	36.8 ± 6.8	0.695
Total body water (%)	55.8 ± 6.2	55.5 ± 5.4	0.987
Phase angle	6.00 ± 0.7	6.00 ± 0.6	0.883

BMI—body mass index.

**Table 6:** Serum Markers in Asymptomatic Subjects at the Baseline and Post-Lockdown.

	Baseline	Post-Lockdown	*p* Value *	Slovenian Reference Values	∆
Glucose (mmol/L) *	4.850 ± 0.425	5.210 ± 0.698	0.005	3.6–6.1	0.361 ± 0.746
Total cholesterol (mmol/L) *	5.447 ± 3.523	5.947 ± 3.445	0.003	4.0–5.2	0.500 ± 0.985
HDL (mmol/L)	2.008 ± 0.402	2.075 ± 0.592	0.375	>1.4	0.067 ± 0.459
LDL (mmol/L) *	3.852 ± 3.614	4.102 ± 3.560	0.049	2.0–3.3	0.250 ± 0.815
Triglycerides (mmol/L)	0.953 ± 0.574	1.138 ± 0.640	0.054	0.6–1.7	0.186 ± 0.679
CRP (mg/L) *	0.941 ± 1.315	0.635 ± 1.108	0.008	0.0–8.0	−0.306 ± 1.591
Iron (μmol/L)	23.263 ± 10.663	25.905 ± 9.850	0.283	M 10.6–28.3, W 6.6–26.0	2.642 ± 11.887
AST (U/L)	22.150 ± 6.606	23.613 ± 10.701	0.658	M < 34.8, W < 31.2	1.463 ± 10.270
Total bilirubin (μmol/L)	8.405 ± 5.369	9.768 ± 5.422	0.066	0.0–17.0	1.363 ± 4.330

* *p* < 0.05 is considered statistically significant; HDL—High-density lipoprotein; LDL—Low-density lipoprotein; CRP—C-reactive protein, AST—Aspartate transaminase; M—men, W—women, ∆—changes of post-lockdown data from baseline.

## Data Availability

The data presented in this study are available on request from the corresponding author.
